# CRISPR/Cas9 nickase-mediated disruption of hepatitis B virus open reading frame S and X

**DOI:** 10.1038/srep13734

**Published:** 2015-09-03

**Authors:** Madina Karimova, Niklas Beschorner, Werner Dammermann, Jan Chemnitz, Daniela Indenbirken, Jan-Hendrik Bockmann, Adam Grundhoff, Stefan Lüth, Frank Buchholz, Julian Schulze zur Wiesch, Joachim Hauber

**Affiliations:** 1 Heinrich Pette Institute – Leibniz Institute for Experimental Virology, 20251 Hamburg, Germany; 2 Department of Medicine I, University Medical Center Eppendorf, 20246 Hamburg, Germany; 3 German Center for Infection Research (DZIF), partner site Hamburg, Hamburg, Germany; 4 Department of Medical Systems Biology, University Hospital and Medical Faculty Carl Gustav Carus, TU Dresden, 01307 Dresden, Germany

## Abstract

Current antiviral therapies cannot cure hepatitis B virus (HBV) infection; successful HBV eradication would require inactivation of the viral genome, which primarily persists in host cells as episomal covalently closed circular DNA (cccDNA) and, to a lesser extent, as chromosomally integrated sequences. However, novel designer enzymes, such as the CRISPR/Cas9 RNA-guided nuclease system, provide technologies for developing advanced therapy strategies that could directly attack the HBV genome. For therapeutic application in humans, such designer nucleases should recognize various HBV genotypes and cause minimal off-target effects. Here, we identified cross-genotype conserved HBV sequences in the S and X region of the HBV genome that were targeted for specific and effective cleavage by a Cas9 nickase. This approach disrupted not only episomal cccDNA and chromosomally integrated HBV target sites in reporter cell lines, but also HBV replication in chronically and *de novo* infected hepatoma cell lines. Our data demonstrate the feasibility of using the CRISPR/Cas9 nickase system for novel therapy strategies aiming to cure HBV infection.

Worldwide, 350 million people are chronically infected with hepatitis B virus (HBV) and are potentially at risk of developing cirrhosis of the liver or hepatocellular carcinoma (HCC)^1^,^2^,^3^. The genome of the enveloped HBV comprises a circular, partially double stranded DNA that is synthesized from an RNA intermediate using a reverse transcriptase^4^,^5^. To date, eight well-known genotypes (A-H) of the HBV genome have been described, each differing in more than 8% of the nucleotides^6^,^7^. In HBV infected cells the genetic information is present both episomally as so-called cccDNA (covalently closed circular DNA), and in the human genome as integrated DNA^4^,^5^,^8^. This viral reservoir makes lasting HBV therapy very difficult. Current therapies for chronic hepatitis B are expensive, can cause serious side effects (e.g. interferon-α therapy) or must be taken for years (e.g. therapies using nucleos(t)ide analogues) with the risk of toxic effects and developing escape mutations of the virus^9^,^10^,^11^.

While HBV treatment leads to full seroconversion in a small minority of patients treated with interferon-α for 48 weeks, or (near) viral suppression in most patients treated with nucleos(t)ide analogues, complete elimination of HBV from the body is not possible with the current therapy principles, since the viral DNA is an integral part of the DNA in liver cells^12^,^13^. A complete cure of HBV infection would require eradication or at least massive reduction of the cccDNA from the infected hepatocytes^14^,^15^. Such an eradication protocol could possibly be used in combination with other methods (e.g. immunomodulation, small-molecular weight antiviral inhibitors, RNAi etc.)^16^,^17^,^18^.

Over the last few years, at least three revolutionary genome editing technologies have been developed, such as zinc finger nucleases (ZFNs), transcription activator-like effector nucleases (TALENs) and, most recently, the CRISPR/Cas9 RNA-guided nuclease system^19^. Indeed, direct antiviral effects against HBV have been achieved in different test systems by using ZFNs^20^,^21^,^22^, TALENs^23^,^24^, and recently CRISPR/Cas9^25^,^26^,^27^,^28^. However, besides optimal delivery of the nucleases^29^, and optimal target site conservation in circulating HBV strains^6^,^7^, there is the highly relevant issue of potential off-target effects^30^,^31^.

Lately, a mutated “nickase” version of the Cas9 enzyme (Cas9n) has been described that introduces a single-strand DNA break (nick) at a specific location based on a co-expressed guiding RNA (gRNA)-defined target sequence, rather than the double-strand DNA breaks (DSBs) produced by the wild-type enzyme^32^,^33^. Thus, by providing Cas9 nickase (Cas9n) together with a pair of properly spaced gRNAs, two adjacent, opposite strand nicks can cause DSBs and trigger error-prone non-homologous end joining (NHEJ) repair. Importantly, undesired off-target nicks are precisely repaired, using the intact strand as a template^32^,^33^. For this reason, the double-nicking approach improves target specificity by up to 1,500-fold compared to the Cas9 wild-type protein^30^,^32^,^34^. This significant improvement of enzyme fidelity is clearly of great interest with respect to future therapeutic application of Cas9 nuclease in humans, for example for curative treatment of chronic HBV infection.

Here, we investigated the potential use of the improved CRISPR/Cas9 nickase system to selectively target and inactivate episomal as well as chromosomally integrated HBV sequences in reporter cells and chronically infected hepatoma cell cultures. We demonstrate that two highly conserved regions in the S and X gene of HBV can be exploited for Cas9n-mediated inactivation of HBV, supporting the notion that further clinical development of engineered designer nucleases may eventually allow the eradication of HBV in infected patients.

## Results

### Identification of conserved target sites in the HBV genome

Genetic diversity is a hallmark feature of hepatitis B virus strains, which exist as eight distinct genotypes (A-H) distributed around the globe^6^,^7^,^35^. To potentially target a majority of HBV isolates by Cas9n, we first identified conserved sequences in the viral genome. A publicly available database of HBV isolate sequences was used to analyze sequence conservation^36^. First, using a total of 1,931 genotype A sequences, the HBV genome was analyzed to find a region with a length of at least 50 nucleotides, where according to the alignments dataset, the individual positions have high conservation values (more than 99.9%).

**Figure 1:**
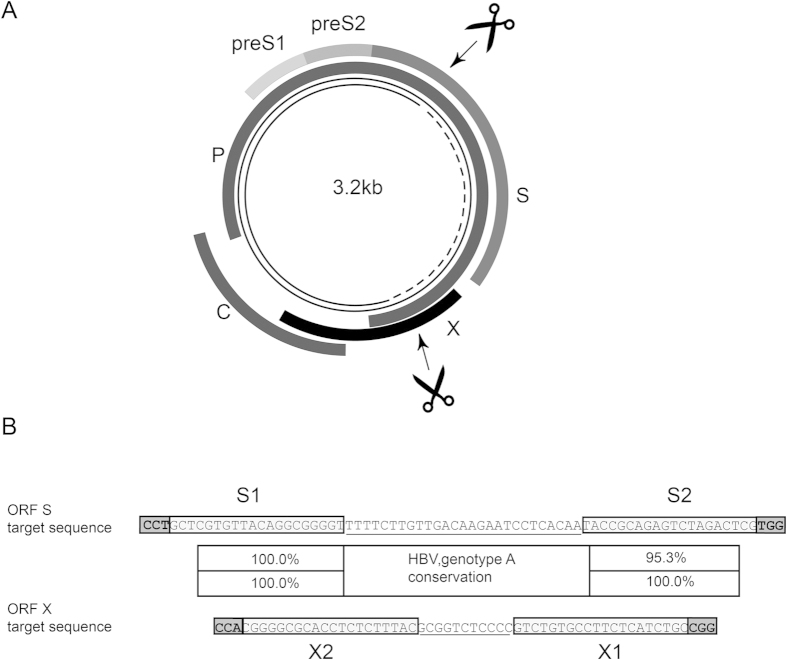
HBV-specific gRNA target sites for Cas9n recruitment. (**A**) Schematic representation of the hepatitis B virus genome. Relaxed circular DNA (rcDNA) of the HB virion (thin continuous line), which is converted to cccDNA (thin continuous and dotted line) following hepatocyte infection, is indicated in the centre of the map. The four viral transcripts of the core (C), polymerase (P), and surface (S) and X proteins are indicated around the outside. Regions targeted by Cas9n via guide RNA (gRNA) specific to S and X sequences are indicated by arrows and scissors (scissors were drawn by Niklas Beschorner). (**B**) DNA sequence and sequence conservation of the regions targeted by Cas9n within the S and X gene of HBV. The sequence shown is based on genotype A consensus. Target sequences in ORF S and X are depicted (S1 and S2, or X1 and X2), each encompassing proto-spacer adjacent motifs (PAM, bold and boxed), 2 × 20 nucleotides complementary to gRNA (boxed) and offset distance between the two sequences complementary to gRNA (underlined).

Two conserved regions were identified within the open reading frame (ORF) S and X (each overlapping with ORF P) of the HBV genome (indicated by scissor symbols in Fig. 1A). In each of these conserved sequences, a single pair of highly conserved single guide RNA (sgRNA) Cas9n target sequences plus proto-spacer adjacent motifs (PAM) was identified (referred to as S1 and S2, or X1 and X2) which fulfilled all the filtering criteria, i.e. displayed >95% conservation over the full sequence of 23 nucleotides (nt) among the 1,931 genotype A isolates (for details see Methods section and Fig. 1B).

The possibility of using these conserved regions for potentially inactivating other genotypes was subsequently evaluated by calculating the conservation of the respective 23 nt target sequence among isolates belonging to B, C, D, E, F and G genotypes. The sgRNA target sequences S1, S2, X1 and X2 exhibit more than 93% conservation over the full 23 nt sequence among the analyzed isolates of the B, C, and E genotype, and more than 89% conservation over the respective genotype D sequences, indicating high sequence conservation between HBV genotypes differing in their geographic distribution (Supplementary Table 1).

### Functional validation of potential target sites located in the open reading frame S and X

For functional analyses, the selected 20 bp gRNA sequences were individually cloned into expression plasmids expressing human codon-optimized Cas9n (D10A), with a pol III (U6) promoter driving single guide RNA (sgRNA) expression, as a 20 nt gRNA and *trans*-activating CRISPR RNA (tracrRNA) fusion^32^,^33^.

**Figure 2:**
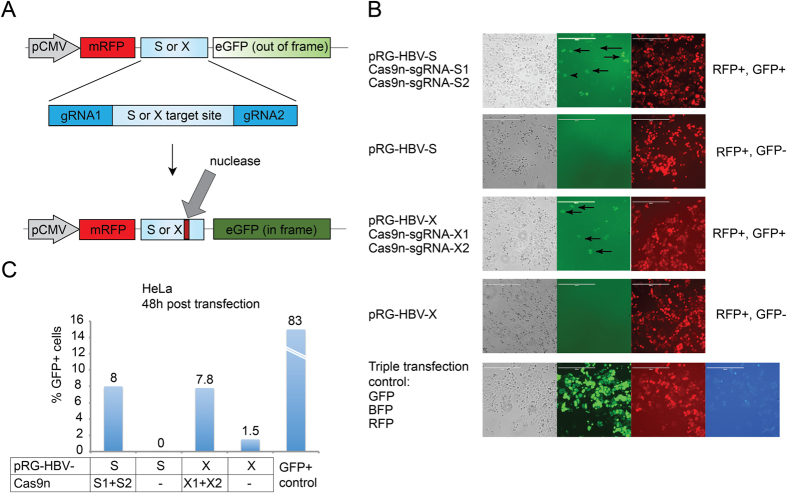
Target site validation by transient transfection of HeLa cells. (**A**) The HBV reporter plasmid for detecting Cas9n nuclease activity on HBV S or X target sequences is depicted at the top. The reporter construct contains a constitutive CMV promoter and sequences encoding RFP (red fluorescent protein) and GFP (enhanced green fluorescent protein), the latter lacking a start codon and positioned out of frame. RFP and GFP sequences are separated by the HBV S or X target site. Each target sequence contains two 20 bp regions necessary for gRNA binding and PAM motifs (expanded region). Cas9n nuclease activity aided by the pair of sgRNAs leads to two individual single stranded breaks within the target sequence (indicated by the large arrow; Cas9n cleaves the target sequence 3 nt upstream of the PAM region) which, upon non-homologous end joining repair, can lead to subtle sequence deletions and thereby frame shifts of the downstream GFP-specific sequence. (**B**) Cas9n activity on HBV sequences in HeLa cells at 48 h post transfection. Cultures were transfected with HBV S- or X-specific reporter and two Cas9n/sgRNA expression plasmids; HBV reporter plasmids alone (control); or with vectors expressing RFP, GFP and BFP (transfection control). Arrows indicate GFP expressing cells due to Cas9n activity. Scale bar = 400 μm. (**C**) Percentage of the total GFP + population in cultures containing the indicated vectors. Transfection control measured the presence of the positive control plasmid (GFP  +) by flow cytometry.

After identifying potential gRNA target sequences, pRG-HBV double fluorescent reporter constructs were generated to detect Cas9n activity. The vector design was based on comparable constructs previously reported by Kim and coworkers^37^. The pRG-HBV reporter plasmid constitutively expresses RFP and also contains the respective HBV S or X gene target sequence (73 bp or 57 bp in length; Fig. 1B) as well as the gene encoding eGFP, which is positioned out of frame relative to the RFP sequence (depicted in Fig. 2A). As indicated, the S- and X-specific sequences contain two 20 bp regions with adjacent PAMs necessary for gRNA binding and subsequent Cas9n-mediated double nicking. Thus, recruitment of Cas9n activity to the respective HBV-specific target sequence by two properly spaced sgRNAs results in a double strand break (indicated by an arrow in Fig. 2A), which, upon NHEJ repair, leads to indel formation. Due to codon triplet usage, statistically 1 out of 3 repairs then results in an “in frame” fusion of the eGFP gene to the mRFP gene located upstream of the cleavage site. Therefore, the presence of the reporter is detected by a sole RFP signal, while nuclease activity is visualized by simultaneous GFP and RFP fluorescence.

A set of three plasmids comprising the pRG-HBV-S or pRG-HBV-X reporter construct together with two appropriate Cas9n/sgRNA expression plasmids was used to monitor Cas9n activity on the respective HBV sequences in human cells. In addition, transfection with the HBV reporter plasmids alone (i.e. omitting Cas9n/sgRNA expression constructs) or cotransfection with individual plasmids for RFP, GFP or BFP expression served as controls. Cas9n activity was detected in HeLa cells at 48 hours post transfection (Fig. 2B, green panels). As expected, cell cultures containing just the HBV S or X reporter construct did not display any GFP signals but were positive for RFP expression. In contrast, GFP signals were detected in cells cotransfected with vectors expressing Cas9n and sgRNAs along with the matching HBV reporter (arrows in Fig. 2B), indicating that in the respective target sequence Cas9n/sgRNA-mediated cleavage followed by NHEJ repair had occurred, positioning the sequences encoding GFP and RFP in frame with each other.

Another transfection of HeLa cells was used to quantify Cas9n activity via FACS analysis. As depicted, transfected cultures containing the pRG-HBV-S or pRG-HBV-X reporter construct either failed or displayed only a very low amount of cells positive for GFP expression; i.e. 0% and 1.5 % of GFP + cells (Fig. 2C), which is consistent with the data seen by fluorescence microscopy (Fig. 2B). However, when vectors for Cas9n and sgRNAs were cotransfected along with the reporter plasmids, clearly elevated levels of fluorescence were detected at 48 hours post transfection, with 8% of GFP + cells in the culture where HBV S sequences were the target, and 7.8% of GFP + cells in the culture where X sequences were recognized (Fig. 2C).

**Figure 3:**
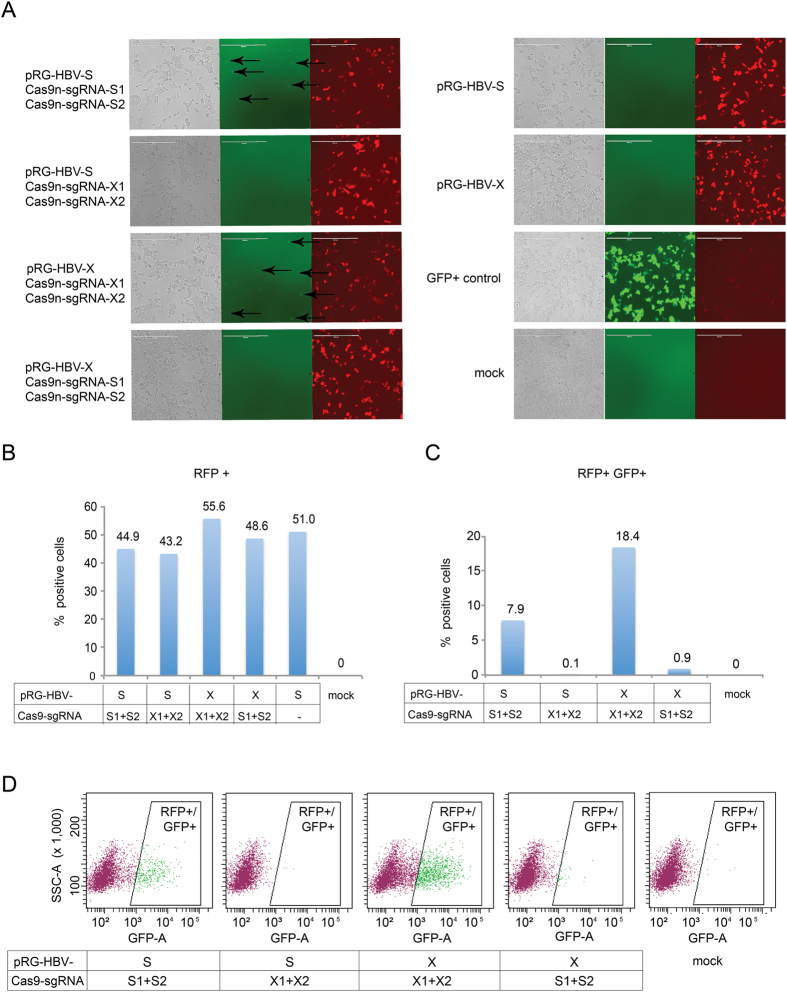
Analysis of Cas9n activity in HEK293 cells. (**A**) HEK293 cells were transfected with the respective HBV reporter (pRG-HBV-S or pRG-HBV-X) and two plasmids for Cas9n/sgRNA expression (Cas9n-sgRNA-S1 and Cas9n-sgRNA-S2; Cas9n-sgRNA-X1 and Cas9n-sgRNA-X2). For transfection control, cells were transfected with a constitutively GFP-expressing plasmid or mock-transfected. Cells were imaged at 24 h post transfection. Arrows indicate GFP expressing cells due to Cas9n activity. Scale bar = 400 μm. (**B**) Cells were analyzed by FACS for RFP fluorescence to determine the presence of the HBV reporter in the transfected cells. (**C,D**) Target-specific Cas9n/gRNA nuclease activity was quantified by FACS analysis of GFP + cells within the populations of RFP + (reporter containing) cells.

To analyze Cas9n activity on HBV sequences in another cell line, we next transfected and quantified HEK293 cells as above. For a control of specificity we now included transfections with mismatched reporter and Cas9n/sgRNA expression plasmids. Similar to the previous results, at 24 hours post transfection GFP was only successfully induced in the cell cultures cotransfected with HBV reporters and the vectors expressing Cas9n and their matching sgRNAs (Fig. 3A; arrows). The control experiments, constitutively expressing GFP, containing reporter vector only, or containing mismatched pairs of reporter and sgRNAs, revealed the expected phenotypes (Fig. 3A). Furthermore, FACS analyses demonstrated that the number of RFP + positive (i.e. transfected) cells was comparable in each HEK293 culture (Fig. 3B), while the number of RFP + /GFP + cells substantially increased in the cultures transfected with matching pairs of reporter and Cas9n/sgRNA expression plasmids (Fig. 3C,D).

**Figure 4:**
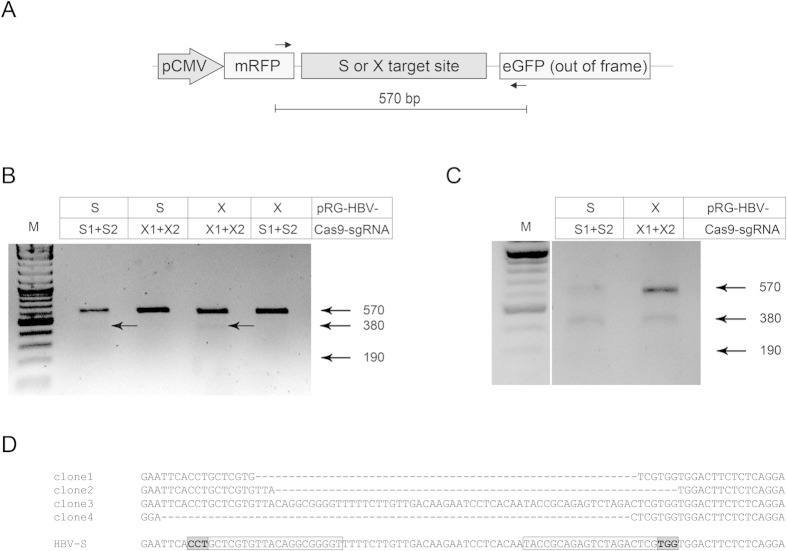
Analysis of Cas9n activity by T7 endonuclease I assay. (**A**) T7EI assays were performed using PCR primers (indicated by arrows) flanking the HBV S or X sequence in the respective reporter plasmid. (**B**) Detection of Cas9n-specific activity was visualized by gel electrophoresis. HEK293 cells were transfected as before and total genomic DNA was isolated at 72 h post transfection for subsequent T7EI cleavage. Arrows depict the sizes of wild-type and Cas9n-mutagenized DNA fragments. (**C**) T7EI assay using genomic DNA from GFP + HEK293 cell cultures at 24 h post transfection. (**D**) Sequence analysis of corresponding DNA samples. Alignment to the wild-type ORF S reporter sequence is shown. gRNA sequences (boxed), PAM (boxed and bold) and Cas9n-mediated deletions are indicated.

To more directly detect Cas9n-mediated mutagenesis of HBV-derived target sequences, we performed an assay with T7 endonuclease I (T7EI), which detects Cas9-induced mutations by cutting the DNA at mismatched nucleotides^37^,^38^. We transfected HEK293 cells as before and obtained total (chromosomal and episomal) cellular DNA from sorted GFP + cells, which served as templates for PCR-mediated enrichment of the HBV S or X target sequences (Fig. 4A). Following the T7EI assay, smaller sized products of approximately 380 and 190 bp, indicating the presence of mismatched DNA in the target sequence, were only present in cell samples transfected with the correct combination of reporter and Cas9n/sgRNA expression plasmids (Fig. 4B,C).

To verify indel formation in the HBV reporter we performed a straightforward sequence analysis. HEK293 cells were cotransfected as before with pRG-HBV-S reporter and matching Cas9n/sgRNA expression plasmids. Total DNA was isolated from RFP + /GFP + cells, transformed into *E.coli* and individual HBV sgRNA target sites were analyzed in selected recovered reporter plasmids. As an example, of the four sequences shown in Fig. 4D, three represent Cas9n/sgRNA-induced deletions (individual clones #1, #2, and #4; Fig. 4D). Of note, Cas9n cleaves the target sequence 3 nt upstream of the PAM region (Fig. 4D; PAM indicated in bold letters). Since the HBV S-specific sgRNAs were designed to anneal to opposite strands with an offset of 27 nt, subsequent cleavage creates single-stranded 5′ overhangs, which are prone to deletion by NHEJ. Therefore, the observed deletions represent almost perfect 5′ overhang sequences (Fig. 4D).

Taken together, these data suggest that sequences in the HBV S and X gene can be recognized in human cells by sgRNAs and serve as substrates for subsequent Cas9n-mediated indel formation.

### Cas9n activity on integrated HBV reporter constructs

The persistence of episomal nuclear cccDNA is considered a main obstacle of curative HBV therapies. However, random integration of HBV DNA into the host cell genome is common, which may also contribute to disease outcome^8^. Therefore, the accessibility of stably integrated HBV S and X target sequences for Cas9n was tested.

**Figure 5:**
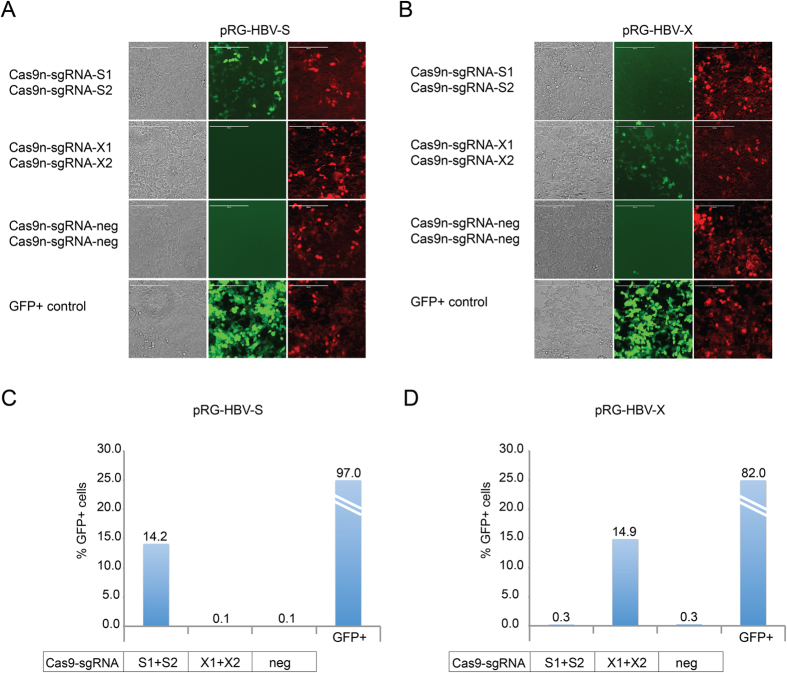
Targeting chromosomally integrated HBV-sequences using the CRISPR/Cas9n system in HEK293 cell cultures. (**A**) Fluorescence microscopy images of the stable HBV S-specific reporter cell line cotransfected with plasmids expressing Cas9n and S or X (control) sequence-specific sgRNAs, or sgRNA targeted to an unrelated locus (negative control; Cas9n-sgRNA-neg). A constitutively GFP-expressing vector served as a transfection control. GFP expressing cells indicate Cas9n activity. Scale bar = 400 μm. (**B**) Analysis of a stable HBV X-specific reporter cell line as above. (**C**) HBV S sequence-specific Cas9n/sgRNA-mediated nuclease activity was quantified by GFP-specific FACS analysis. (**D**) As in C to quantify HBV X sequence-specific Cas9n/sgRNA-mediated nuclease activity.

**Figure 6:**
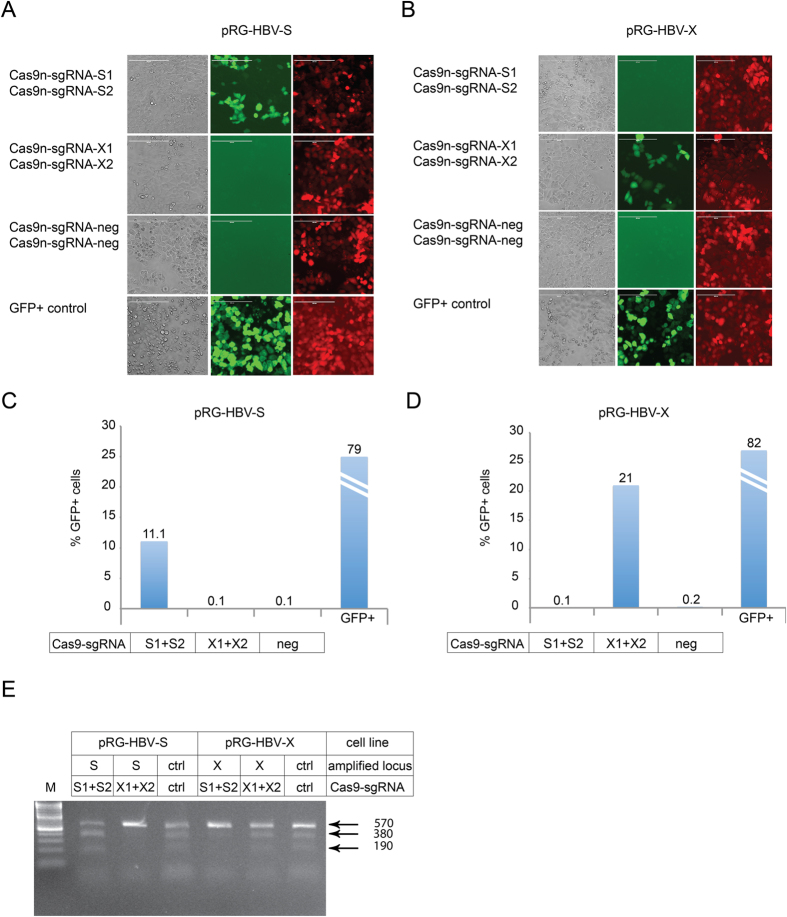
Analysis of integrated HBV reporter constructs in HeLa cells. (**A**) Targeting of chromosomally integrated HBV ORF S sequences by the CRISPR/Cas9n system was analyzed at 72 h post transfection, as described in Fig. 5A. GFP expressing cells indicate Cas9n activity. Scale bar = 400 μm. (**B**) Analysis of chromosomally integrated HBV ORF X sequences as described in Fig. 5B. (**C,D**) Quantification of the experiments shown in panel A and B, respectively, by FACS analysis of GFP + cells. (**E**) The stable HBV S and X target site-specific HeLa cultures, transfected with the indicated combinations of Cas9n and sgRNA-expressing vectors, analyzed by the T7EI cleavage assay. Targeting of an arbitrary genomic locus by sgRNA specific to this region was used as a positive control (ctrl).

Stable HeLa and HEK293 cell lines containing integrated HBV-X or HBV-S reporter sequences (see Supplementary Figure 1) were generated using PiggyBac targeting vectors (System Biosciences Inc.). These cell lines were transfected with matching or mismatching (control) Cas9n/sgRNA expression vectors. Additional control experiments were performed by transfecting negative control sgRNA vectors (targeting an unrelated genomic locus), or a constitutively GFP-expressing plasmid (positive control). At 72 hours post transfection, cultures were analyzed by fluorescence microscopy and FACS as above. In both the HEK293 cell cultures (Fig. 5) and HeLa cell cultures (Fig. 6), the number of GFP + cells only increased in the cultures transfected with plasmids expressing sgRNAs matching the integrated HBV reporter sequences (Figs 5A–D and 6A–D), demonstrating that Cas9n/sgRNAs accurately cleave their targets in a genomic context.

Stable HeLa cells, containing either HBV-S or HBV-X reporter sequences were next analyzed by the T7EI assay (Fig. 6E). An arbitrary genomic locus previously tested for efficient Cas9n targeting, and matching specific sgRNA was used as a positive control (ctrl). The smaller bands, representing T7 endonuclease-cleaved DNA strands where a mismatch was introduced by the Cas9n/sgRNA system, were only present in cell samples transfected with a pair of Cas9n/sgRNA expression plasmids matching the integrated HBV reporter sequence.

These data demonstrated that the Cas9n/sgRNA system can successfully target HBV-derived sequences that are stably integrated into the host cell genome.

### HBV inactivation in chronically and *de novo* infected hepatoma cell lines

At this point, the combined data obtained by using reporter constructs indicated that episomal or integrated sequences encoding the HBs or HBx antigen can be substrates for Cas9n-mediated inactivation. However, future application in curative HBV-therapies requires targeting the HBV genome in chronically infected hepatocytes. We therefore investigated the established hepatocyte cell lines HepG2.2.15 and HepG2-H1.3. Both of these cell lines carry HBV genomes, including chromosomally integrated sequences and cccDNA, and importantly, release virus particles into the culture supernatant^39^,^40^.

**Figure 7:**
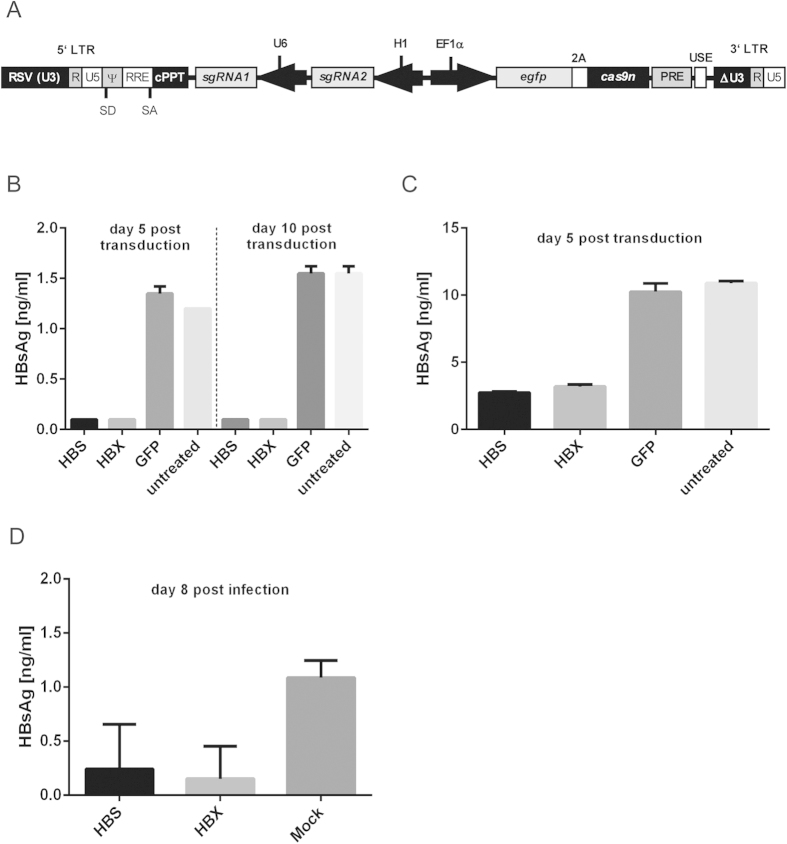
Inactivation of HBV in chronically and *de novo* infected hepatocytes. (**A**) The backbone of the HIV-derived lentiviral vector (LV) for delivering Cas9n and a pair of sgRNAs contains self-inactivating (SIN) long terminal repeats (LTR:ΔU3, R, U5), a Rev response element (RRE), a central polypurine tract (cPPT), a woodchuck hepatitis virus post-regulatory element (PRE), SV40 polyadenylation enhancer elements (USE), splice donor (SD), splice acceptor (SA) and packaging signal (Ψ) sites. Expression of an eGFP-2A peptide-Cas9n fusion protein is regulated by the internal human elongation factor 1α (EF1α) promoter. Transcription of two sgRNAs, sgRNA1 or sgRNA2, is regulated by a U6 or H1 pol III promoter. (**B**,**C**) HBsAg in the filtered supernatants of HepG2.2.15 and HepG2-H1.3 cells was quantified by ELISA at day 5 and/or at day 10 post transduction with LV-HBS, LV-HBX or LV-GFP. Experiments were performed in duplicate. The lower limit of detection (Background + 3 x S.D.) was 0.9 ng/ml for HBsAg. All values are given as mean concentration ng/ml ± S.D. (**D**) HBsAG in the filtered supernatant of LV-HBS, LV-HBX or mock transduced HepG2^hNTCP^ cells was quantified as described before at day 8 post HBV infection. Experiments were performed in quadruplicate.

To transduce the respective cell cultures with expression cassettes for Cas9n and the two target site-specific sgRNAs, we constructed a self-inactivating (SIN) lentiviral vector (LV). Gene sequences encoding eGFP and Cas9n were placed under the control of the human elongation factor-1 α (EF1α) promoter separated by the equine rhinitis A virus (ERAV) 2A-like sequence^41^, which allows the enrichment of transduced cells (i.e. GFP + cells) by FACS. In the opposite transcriptional direction, the pair of sgRNAs required to recruit Cas9n to a specific HBV target site are expressed independently by a U6 and a H1 pol III promoter (Fig. 7A).

HepG2.2.15 cells were transduced with VSV-G pseudotyped Cas9n/sgRNA-expressing LV particles, or a corresponding negative control vector expressing GFP alone (LV-GFP). Transduced hepatocytes were enriched by FACS and release of HBV particles was monitored over time by HBsAg ELISA. While accumulation of HBV in the culture supernatants increased with time in untreated and LV-GFP-transduced cultures, no HBV particle release was detected by HBsAg ELISA from HepG2.2.15 cells transduced with LV expressing Cas9n together with sgRNA specific for either ORF S (HBS) or ORF X (HBX) (Fig. 7B). Similarly, transduction of HepG2-H1.3 cells with LV-HBS or LV-HBX resulted in significant inhibition of HBV progeny formation at day 5 post transduction (Fig. 7C).

Finally, we investigated Cas9n-mediated HBV inactivation in *de novo* infected hepatocytes. HepG2^hNTCP^ cells^42^,^43^ were transduced as before with Cas9n/sgRNA-expressing LV particles and infected with HBV, purified from cell culture supernatant of HepG2.2.15 cells^39^. Again, HBsAg release was clearly impaired at day 8 post infection in cells expressing Cas9n together with ORF S- or X-specific sgRNAs (Fig. 7D), indicating that the viral cccDNA was targeted.

**Figure 8:**
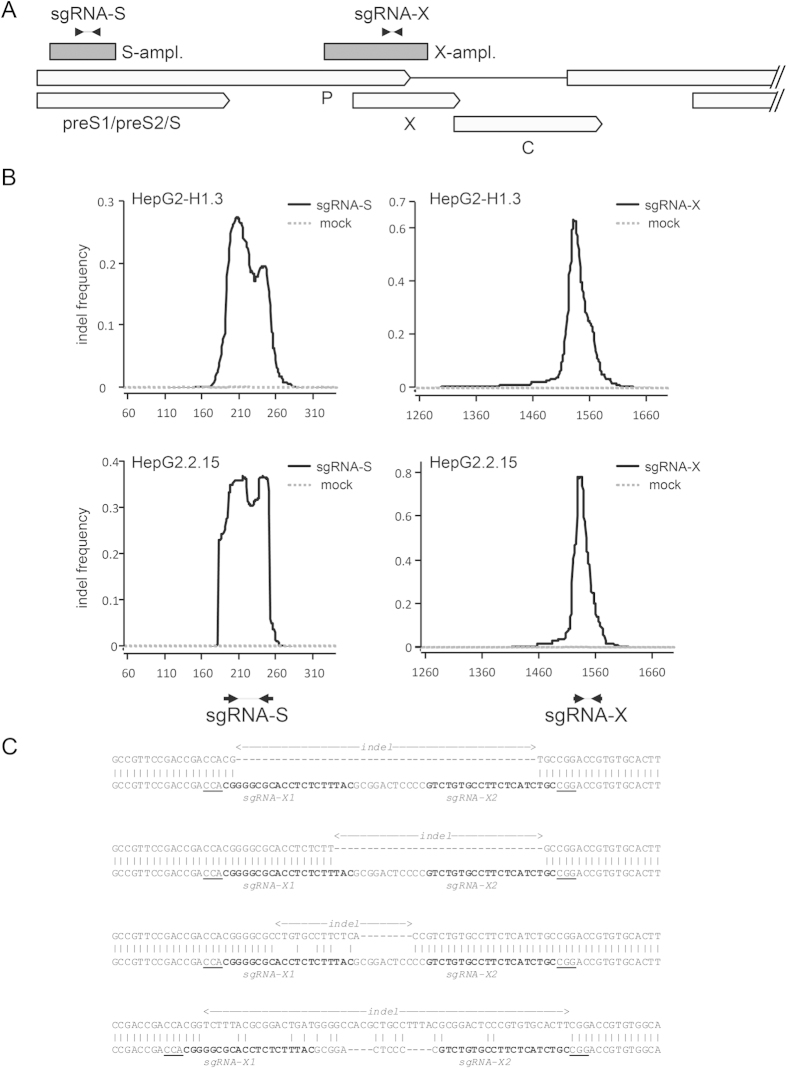
Analysis of indels in sgRNA-treated HepG2-H1.3 and HepG2-H2.2.15 cells. (**A**) Scheme of the HBV genome depicting the location of amplicons spanning the S- and X-specific sgRNA target regions. The two amplicons are shown as dark gray boxes labelled S-ampl. and X-ampl. Arrows shown at the top indicate the genomic location of gRNA target sequences. (**B**) Indel frequency as detected in S-amplicons (left) or X-amplicons (right) from HepG2-H1.3 (top panels) or HepG2.2.15 (bottom panels) cells. The graphs represent the frequency with which each individual amplicon’s nucleotide is affected by indels in sgRNA-expressing (solid black line) or mock transduced cells (gray dashed line). Nucleotide positions given on the x-axis indicate coordinates on the full-length HBV genome (accession JN664938). The locations of gRNA target sequences are indicated by arrows underneath the bottom panels. (**C**) Example alignments of indel amplicon reads from Cas9n/sgRNA-X-expressing HepG2-H1.3 (top sequence in each alignment) aligned to wild-type HBV amplicon sequences (bottom sequence in each alignment). Indel sites are indicated above each read. The sgRNA target sequences and PAM motifs appear in bold or underlined, respectively.

The combined data from these three models of HBV infection suggest that vector-mediated delivery of the Cas9n-system, targeted to virus-specific genomic sites, inactivated persistent virus. To directly confirm this observation, we subjected total cellular DNA from the HepG2.2.15 and HepG2-H1.3 cultures to target-site-specific next generation sequencing. After generating amplicons containing the target regions of S- or X-specific sgRNAs from HepG2.2.15 or HepG2-H1.3 cells, we analyzed approximately 350,000 amplicons from each sample using an Illumina MiSeq instrument. Amplicons from mock-transduced control cultures were sequenced in parallel to establish the wild-type sequences of resident HBV genomes (see Methods section for details). The results confirmed that efficient editing of HBV genomes had occurred in Cas9n/sgRNA expressing cells. On average between 44% and 89% of all reads derived from sgRNA-S- or sgRNA-X-expressing cells, respectively, exhibited indel signatures (Table 1). Consistent with the expected pattern of Cas9n/sgRNA-induced mutations, the spatial distribution of nucleotides affected by the various indels showed a marked peak at sites complementary to S- and X-specific sgRNAs (Fig. 8A,B). Also as expected, no deletions or insertions were detected in amplicons from the mock-infected controls (dashed gray lines in Fig. 8B).

Although indels were generally located at or near gRNA target sites, individual indel events varied with regard to the lengths of deletions, as well as lengths and nucleotide sequences of insertions. The average length of deleted sequences was between 37–51 nucleotides in sgRNA-S treated, and 30–35 nucleotides in sgRNA-X treated cells, while the average length of insertions was approximately 2 to 3 nucleotides in all cases (Table 1). The identified deletions were similar to the nickase-mediated indel lengths observed using the reporter plasmid approach (Fig. 4D). Using the HBV reporter plasmid, Cas9n induced “offset” deletions by cutting 3 nt upstream of the PAM. The predicted “offset” deletions for S (61 nt) and X (45 nt) were observed to some degree in our next generation sequencing samples. However, substantial fractions of reads in each of the samples also showed deletions or insertions with lengths significantly longer or shorter than these average values (for a more detailed representation of length distributions see Supplementary Figures 2 and 3).

Several examples of indel read alignments from Cas9/sgRNA-X transduced HepG2-H1.3 cells are shown in Fig. 8C. The first and second alignments represent contiguous deletions of 46 and 32 nucleotides, respectively, with no additional insertions being present. The third alignment shows a 21 nt deletion together with an insertion of 13 nucleotides, with the latter likely being the result of non-templated addition of nucleotides during NHEJ repair events. Finally, the fourth alignment shows an indel where the number of inserted nucleotides (56 nt) exceeds the length of the primary deletion (48 nt), resulting in a net gain of 8 nucleotides.

## Discussion

The intrinsic stability of the nuclear episomal cccDNA, which serves as transcriptional template for expression of all viral proteins, precludes HBV eradication by current antiviral therapies^44^,^45^. Therefore, novel approaches to eliminate cccDNA are considered the key to cure HBV infection^46^,^47^. Here, we demonstrate that a Cas9n/sgRNA system designed to recognize conserved sequences within the open reading frames S and X of HBV genomes can efficiently inactivate HBV in chronically and *de novo* infected cells.

Future therapies to achieve a sterilizing or at least a functional cure of HBV infection are envisaged to be a combination of different antiviral approaches^46^,^47^,^48^. It is also fair to assume that any such novel treatment strategies will benefit from including technologies that directly target and efficiently inactivate cccDNA. In particular, *in vitro* engineered designer nucleases, such as ZFNs, TALENs or the CRISPR/Cas9 system^19^, appear to be promising components of novel HBV-specific combination therapies. In fact, antiviral effects have been already demonstrated for ZFNs^20^,^21^,^22^, TALENs^23^,^24^, and CRISPR/Cas9^25^,^26^,^27^,^28^ in various HBV model systems. Indeed, the RNA-guided CRISPR/Cas9 nuclease system appears to be particularly attractive, since generating novel guide RNAs for Cas9 targeting to HBV genomic sequences is much faster than the laborious construction of target-specific ZFNs or TALENs. Moreover, by providing multiple guide sequences CRISPR/Cas systems permit multiplexed genome engineering by simultaneous editing of various target sites^49^, an advantage that in some cases might be required to quantitatively eliminate persistent viruses.

A caveat of the CRISPR/Cas9 system could be the fact that relying on a single guiding RNA of ~ 20 nucleotides for targeting Cas9 nuclease to a specific genomic site may be hampered by multiple off-target cleavage^30^,^31^,^50^,^51^,^52^. Obviously, this significantly limits the therapeutic application of Cas9-mediated gene modification in humans. To demonstrate the applicability and specificity of the CRISPR/Cas9 RNA-guided nuclease system in HBV infection, we decided to investigate a potential double-nickase approach. As shown previously, a pair of appropriately spaced Cas9 nickase mutants will introduce two single-strand breaks on opposite DNA strands, which are subject to NHEJ repair^32^,^33^. This approach avoids off-target mutations by improving specificity by up to 1,500-fold relative to the wild-type Cas9 enzyme^30^,^32^,^53^,^54^.

The use of tailored designer nucleases to eradicate HBV cccDNA is, to some extent, complicated by the fact that this common virus possesses pronounced genetic diversity, existing as eight distinct genotypes globally^6^,^7^,^35^. Thus, for potential clinical application one needs to identify not only suitable Cas9 target sites (i.e. displaying correct PAM sequences), but also virus-specific sequences that are maximally conserved between various HBV genotypes. Fortunately, by analyzing a comprehensive database of HBV sequences^36^ we were able to pinpoint two such genomic sites, located in the coding regions for HBs and HBx antigens (Fig. 1B). Our functional analyses subsequently demonstrated that these sites are indeed “drugable” by the CRISPR/Cas9n system.

The use of HBV-specific reporter constructs (Figs 2, 3, 4, 5, 6) as well as infected hepatocytes (Fig. 7) similarly demonstrated pronounced Cas9n effects by targeting either HBV ORF S or X. The failure to detect significant release of HBsAg from hepatocytes where the S or X gene was targeted may be explained by the fact that the respective target sites in the HBV genome also affect the ORF encoding the viral polymerase (Fig. 1A). It is noted that Cas9n-mediated DNA modification was also observed when these target sites were integrated into the host cell genome (Figs 5 and 6). This could be an important point with respect to minimizing the risk of developing HCC, since most HBV integration events retain the ORF encoding HBxAg^8^,^55^.

Analysis of Cas9n activity on ORF S and X target sites by next generation sequencing revealed efficient editing of cccDNA molecules targeted by either gRNA pair in HBV-infected HepG2.2.15 or HepG2-H1.3 cells. The efficiency of X-specific sgRNAs was particularly high: approximately 90% of all amplicons reads showed clearly discernible indel signatures. Although the frequency of indel reads in sgRNA-S targeted cells was lower, our results still suggests that more than 40% of HBV genomes were subject to Cas9n editing. Given that our analysis was based on sequencing amplicons, as opposed to sequencing total DNA moieties, an important question is to what extent these results represent a faithful and quantitative measure of primary editing efficiency. While it is notoriously difficult to determine PCR bias during amplicon sequencing, the great diversity of observed indel events facilitates at least estimating the potential overrepresentation of reads arising from a single editing event. We accordingly calculated the average duplication level of reads with identical indel event signatures containing 10 or more inserted nucleotides; due to the mostly random nature of inserted sequences, these events provide more discriminatory power than simple deletions. The observed duplication levels were generally low (4.2 and 2.0 in sgRNA-S or sgRNA-X expressing cells, respectively; data not shown), suggesting that any bias introduced during PCR is likely to be minor. Furthermore, since the average size of indel reads is only slightly smaller than that of wild-type amplicons, we believe that the numbers given in Table 1 represent a fairly accurate assessment of the percentage of cccDNA molecules edited by Cas9n.

In the proof-of-concept study presented here the CRISPR/Cas9n system was delivered into various human cell lines by lentiviral SIN vector-based gene transfer. Clearly, for clinical application other delivery vehicles, providing a high vector to target cell ratio^56^, should be considered. Since HBV infection is primarily confined to a single organ, the liver, non-integrating vectors that reach high titres, such as adeno-associated virus (AAV) vectors^57^,^58^ or adenoviral vectors^59^,^60^, may be suitable tools for direct and repeated delivery of the CRISPR/Cas9n nuclease system to achieve HBV clearance over time.

In summary, the data presented here support the notion that the CRISPR/Cas9n technology can be a valuable component of advanced combination therapies for eradicating episomal and integrated HBV DNA in infected patients. This system may even provide a strategy for a sterilizing cure of chronic HBV infection.

## Methods

### Target site analysis

The HBV S and X gene was analyzed for the presence of potential gRNA target sites displaying adjacent PAM regions (first strand: 5′-CCN-20 nt gRNA-3′; complementary second strand: 3′-20 nt gRNA-NGG-5′), i.e. for sequences with two PAM motifs in reverse complementary orientation, positioned by an offset of at least 10 bp but not further than 100 bp. Individual pairs of respective gRNA + PAM sequences were analyzed for off-target potential using appropriate software on the http://crispr.mit.edu web resource. Only pairs in which the closest off-target sequence contained at least 3 mismatches in the 20 bp gRNA region were selected for further analysis.

Filtered 23 nt gRNA + PAM sequences underwent a second round of analysis, calculating the nucleotide conservation of the entire 23 nt sequence for each gRNA candidate among 1,931 HBV genotype A isolates^36^. For that, first the consensus sequence of these isolates was used as reference (Supplementary Figure 4 and 5). Frequencies of the semi-conserved positions, i.e. positions where mutation occurs in more than 0.01% of genotype A isolates, were extracted. Only gRNA that spanned the region with less than 2 semi-conserved positions were used for further analysis. Conservation of the gRNA + PAM was calculated by extracting the frequency of the nucleotide at the semi-conserved position (e.g. in the G/A semi-conserved position in gRNA-S1 a guanine appears with a frequency of 95.3% in HBV genotype A) and assigning it as a conservation value for the target sequence. If more than two semi-conserved positions occurred at the same position within the 23 nt sequence, the values were calculated by multiplying two probabilities and extracting the square root. All gRNA + PAM sequences with an overall conservation value higher than 95% were considered as being highly conserved.

The possibility of using these conserved regions for the inactivation of other HBV genotypes was also evaluated by calculating the conservation of the 23 nt sequences among different sequences of genotypes B, C, D, E, F and G^36^. Results are presented in Supplementary Table 1.

### Expression plasmids and lentiviral vector

Cas9n/sgRNA expression vectors were constructed based on the parental pX335 plasmid^49^, which contains a U6 pol III promoter driving expression of the sgRNA (gRNA/tracrRNA fusion), a chicken beta-actin human codon-optimized promoter (CBh) and the bovine growth hormone polyadenylation signal (bGHpolyA) to express Cas9 nickase (human codon-optimized Cas9n D10A mutant fused with two nuclear localization signals). The various sgRNA sequences were constructed for each target separately. For this purpose, complementary oligonucleotides containing a guanine nucleotide necessary for pol III-driven expression and the 20 nt of the corresponding gRNA (targeting HBV S or X sequences) were custom synthesized (biomers.net GmbH) and annealed *in vitro.* Subsequently, the annealed oligonucleotides were ligated into the pX335 plasmid^49^ via BbsI sites. Thus, each of the resulting four expression plasmids contained sequences encoding Cas9n and a single sgRNA (e.g. Cas9n-S1, Cas9n-S2, Cas9n-X1, or Cas9n-X2).

The RFP-GFP reporter plasmids were constructed according to Kim and coworkers^37^. The plasmid backbone was provided by the pEGFP-neo-GFP plasmid (Clontech). First, mRFP was amplified from pDSV-mRFP^61^ and the PCR-product was inserted into the NheI site in pEGFP-N1 (Clontech), which positions the mRFP sequence downstream of a CMV-IE promoter. Subsequently, a cDNA encoding eGFP without a start codon was amplified and ligated between the BamHI and NotI site of this plasmid. Finally, double-stranded oligonucleotides (biomers.net GmbH), containing HBV-derived S or X target sites, were ligated between the mRFP and eGFP sequences exploiting vector-derived EcoRI and BamHI sites. In-frame stop codons were avoided or reduced in the HBV target sequence by changing the orientation of the respective target site.

The backbone of the HIV-derived lentiviral vector for Cas9n and sgRNA expression has previously been described in detail^62^. In brief, the vector contains self-inactivating (SIN) long terminal repeats (LTR: ∆U3, R, U5), a Rev response element (RRE), central polypurine tract (cPPT), transgene cassette, postregulatory element derived from woodchuck hepatitis virus (PRE), SV40 upstream polyadenylation enhancer elements (USE), splice donor (SD), splice acceptor (SA), and packaging (Ψ) signal, and the open reading frame for a GFP-2A peptide-Cas9n fusion RNA under the control of an EF1α promoter. In the opposite transcriptional orientation, two independent sgRNA cassettes are regulated by a pol III H1 or U6 promoter.

Lentiviral particles for infecting cultured cells were produced by transient cotransfection of 293T cells (ATCC catalogue #CRL-3216) with the lentiviral vector construct and the respective packaging plasmids^63^ using *Trans*IT® transfection reagent according to the manufacturer’s protocol (Mirus Bio LCC): specifically, 6 μg of lentiviral vector, 1.5 μg of pRSV-Rev^63^, 1.5 μg of pCMV-VSV-G^64^ and 3 μg of pMDLg/pRRE^63^. At 72 hours post transfection viral supernatants were collected and passed through 0.2 mm pore size filters to ensure removal of any viral aggregates. Titres of lentiviral particles were determined as fluorescent forming units per ml (ffu/ml). This involved transducing 293T cells with different volumes of viral supernatant in the presence of 5 μg/ml protamine sulphate and spinoculation at 300 × g for 10 min at ambient temperature as described in detail^62^. At 72 hours post transduction cells were harvested and analyzed by flow cytometry for GFP expression. Samples that contained 5 to 25% GFP positive cells were used to calculate viral titres.

### Cell culture, transfections and transduction

HeLa cells (CLS Cell Lines Service catalogue #300670) and HEK293 cells (ATCC catalogue #CRL-1573) were plated at a density of 1 × 10^5^ and 2 × 10^5^ cells per well in 12-well dishes, respectively, and grown in DMEM (high glucose), supplemented with 10% (v/v) FBS (Invitrogen) and antibiotics (100 μg/ml penicillin and μg/ml streptomycin). Cultures were grown to ~ 70–80% confluence and subsequently transfected with a total of 1.5 μg plasmid DNA (i.e. RFP-GFP reporter constructs together with the expression plasmids Cas9n/sgRNA-S1 and Cas9n/sgRNA-S2, or Cas9n/sgRNA-X1 and Cas9n/sgRNA-X2, respectively) using Lipofectamin 2000 (Invitrogen) according to the manufacturer’s protocol. Cultures were analyzed by fluorescence microscopy and/or FACS at 24 h, 48 h or 72 h post transfection.

HepG2.2.15 cells^39^ and HepG2-H1.3 cells^40^, human hepatoma cells stably transfected with an HBV 2-fold or 1.3-fold overlength genome, respectively, were grown on rat collagen type I-coated dishes (Serva) in Dulbecco’s modified Eagle medium (high glucose) supplemented with 10% (v/v) FCS (Gibco/Life Technologies), 100 U/ml of penicillin and 100 μg/ml of streptomycin (Life Technologies), 1% (v/v) 0.1 mM non-essential amino acids (Life Technologies), 1% (v/v) 2 mM L-glutamine (Life Technologies), and 1% (v/v) 1 mM sodium pyruvate (Life Technologies). Both cell lines were transduced with various amounts of lentiviral supernatant in the presence of 5 μg/ml protamine sulphate (Sigma-Aldrich) and spinoculated at 300 × g for 10 min at ambient temperature.

Cells were seeded in 24-well plate (TPP, P 92024) coated with rat collagen type I (#47254.01, Serva), supernatant was collected every 5 days, passed through a 0.22 μm Millex® GP filter (Merck Millipore) and stored at −20 °C. Cells were split 1:2 with 0.05% (v/v) trypsin/EDTA (Biochrom, L2153) and transferred to a new plate coated with collagen. Remaining cells were stored as a pellet at −80 °C.

HepG2^hNTCP^ cells^42^,^43^, overexpressing the sodium taurocholate cotransporting polypeptide (NTCP) gene, were cultured and transduced as described above, with the exception, that the culture medium was supplemented with 10 μg/ml Blasticidin-S (*In vivo* Gen).

### HBV preparation and infection

For infection, a 200-fold concentrated culture supernatant of HepG2.2.15 cells was used^39^. Freshly collected supernatant was concentrated with the help of Centricon® Plus-70 Centrifugal Filter Units (Millipore, Darmstadt) and aliquots were stored at −80 °C. A concentration of 1.38 × 10^10^ genome equivalents per milliliter was determined by dot blot. HepG2^hNTCP^ cells were infected using 500 genome equivalents (GEq) per cell. All infections were carried out in the presence of 5% (w/v) polyethylene glycol (PEG) 8000 at 37 °C for 20 h in William’s E differentiation medium (Life Technologies), supplemented with 10% (v/v) FCS (Hyclone, Thermo Scientific), 100 U/ml of penicillin and 100 μg/ml of streptomycin (Life Technologies), 1% (v/v) 2 mM L-glutamine (Life Technologies), 1% (v/v) 1 mM sodium pyruvate (Life Technologies), 0,023 IE/ml human insulin (Sanofi Aventis), 4,7 μg/ml hydrocortisone (Pfizer), 80 μg/ml Gentamicin (Ratiopharm), and 1,8% (w/v) DMSO (Sigma Aldrich). At the end of the incubation period, cells were washed three times with PBS and maintained in William’s E differentiation medium, supernatants were collected every 4 days and stored at −20 °C.

### FACS analysis

HeLa and HEK293 cells were analysed for percentage of GFP + cells using a BD FACSCalibur™ system and CellQuest™ software (Becton Dickinson). Assays were performed in triplicates.

Enrichment of lentiviral vector-transduced HepG2.2.15 and HepG2-H1.3 cells was performed at week 1 and 2 after transduction. Cells were sorted for GFP-positive cells using a BD FACSAria™ Fusion system and FACSDiva™ software (Becton Dickinson).

### T7 endonuclease I assay

For T7 assays (T7EI assay)^37^,^38^ total cellular DNA, comprising chromosomally integrated HBV sequences as well as episomal cccDNA, was isolated using the QIAmp DNA blood kit (Qiagen)^65^,^66^. Regions containing the Cas9-nickase mediated mutations were amplified using Phusion® polymerase (NEB) according to the manufacturer’s protocol with 100 ng of the purified total cellular DNA in a 50 μl reaction. Amplification products were isolated using a PCR purification kit (Qiagen). 400 ng of the purified PCR product was denaturated and re-annealed in a final volume of 10 μl in 1x NEBuffer2 (NEB) using a thermocycler with the following protocol: 95 °C, 5 min; 95–85 °C at −2 °C/s; 85–25 °C at −1 °C/s; hold at 4 °C. Hybridized PCR products were treated with 10 U of T7E1 enzyme (NEB) at 37 °C for 20 min in a reaction volume of 20 μl. An aliquot of 10 μl (200 ng) of the reaction was subsequently analysed on a 3% agarose gel containing ethidium bromide (Sigma Aldrich).

### Analysis of HBV replication

HepG2.2.15 and HepG2-H1.3 cell culture supernatants were passed through 0.22 μm Millex® GP filter (Merck Millipore) and assayed for HBsAg by ELISA (Alpha Diagnostic International) according to the manufacturer’s instructions. Samples were analyzed on a Tecan M200 plate reader using Magellan 6.5 software (Tecan).

### Next generation sequencing

In two consecutive PCR rounds HBV sequences were amplified from HBV-infected cells and adapters for next generation sequencing (NGS) were attached. The primers for the first reaction contained S- and X-specific sequences as well as Illumina-compatible adapters (capital letters): Sfwd: 5′-ACACTCTTTCCCTACACGACGCTCT TCCGATCTcctgctggtggctccagttc-3′; Srev: 5′-TGACTGGAGTTCAGACGTGTGCTCTTCCGATCTttggtgagtgattggaggttg-3′; Xfwd: 5′-ACACTCTTTCCCTACACGACGCTCT TCCGATCTgctcctctgccgatccatac-3′; Xrev: 5′-TGACTGGAGTTCAGACGTGTGCTCT TCCGATCTaggtcggtcgttgacattg-3′. PCR using 250 ng template DNA was performed under the following conditions: 1 cycle: 98 °C for 30 sec–35 cycles: 98 °C for 5 sec/68 °C for 10 sec/72 °C for 10 sec–1cycle: 72 °C for 5 min using Phusion® polymerase (NEB).

A second PCR was performed to extend the Illumina-adapter sequences using a universal 5′-primer and a sample-specific 6-nucleotide barcode (underlined). fwd: 5′-AATGATACGGCGACCACCGAGATCTACACTCTTTCCCTACACGAC-3′; rev: 5′-C AAGCAGAAGACGGCATACGAGATNNNNNNGTGACTGGAGTTCAGACGTGTG-3′. Amplicons were purified after agarose gel electrophoresis using the QIAquick Gel Extraction kit (Qiagen). Diluted libraries (2 nM) were multiplex-sequenced on the Illumina MiSeq instrument. For each sample between 0.62 and 1.1 M paired-end reads of length 2 × 251 bp were generated.

### Bioinformatic analysis of next generation sequencing data

Primary read mapping was performed using CLC Genomics Workbench (v7.5, Qiagen) after merging paired-end reads covering the entire S- or X-amplicons. To establish wild-type HBV sequences, we first mapped reads from untreated HepG2.2.15 and HepG2-H1.3 cells to a reference HBV genome (accession number JN664938) and extracted the consensus sequences for S- and X-amplicons (average coverage between 80,000 and 160,000). To evaluate indels in Cas9n/sgRNA-expressing cells, merged reads were mapped to the consensus wild-type amplicon sequences using the large gap read mapping tool of the transcript discovery plugin (v2.0) of CLC Genomics Workbench. Since Cas9n/sgRNA-directed processing may result in deletions and/or insertions of varying lengths, all SAM alignments containing at least one mismatch, deletion or insertion (PHRED score cutoff >= 20) were considered as potential evidence of template processing. Continuous stretches of non-aligned nucleotides separated by anchored blocks of at least 10 aligned and matched nucleotides were defined as primary processing events.

To generate the indel frequency plots (as shown in Fig. 8) we calculated the frequency of reads that contained processing events affecting each individual nucleotide position in the reference amplicons. For statistical analyses of insertion and deletion lengths as shown in Table 1 and Supplementary Figures 2 and 3, we furthermore calculated independent values for the number of deleted and inserted nucleotides (i.e. the length of reference or read sequences located between aligned anchor blocks) for each individual read. Deletion and insertion values of indel events across individual reads were subsequently summed and a net gain or loss value was calculated by subtracting the total number of deleted nucleotides from the total number of inserted nucleotides. To exclude false positive results due to small mutations introduced during the PCR step of the library preparation, only indel events with a combined length of at least 3 deleted and/or inserted nucleotides were included in the analysis.

Raw sequencing data of all samples have been submitted to the European Nucleotide Archive (ENA) and will be publicly available at http://www.ebi.ac.uk/ena/data/view.

## Additional Information

**How to cite this article**: Karimova, M. *et al.* CRISPR/Cas9 nickase-mediated disruption of hepatitis B virus open reading frame S and X. *Sci. Rep.* **5**, 13734; doi: 10.1038/srep13734 (2015).

## Supplementary Material

Supplementary Information

## Figures and Tables

**Table 1:** Indel statistics.

sgRNA	cell line	total amplicons	amplicons w/indels	% amplicons w/indels	mean deletion length^1^	mean insertion length^2^	mean loss/gain^3^
S	HepG2-H1.3	346,079	142,287	41.1%	36.8	2.4	−34.5
S	HepG2.2.15	353,559	164,468	46.5%	50.7	2.2	−48.5
X	HepG2-H1.3	268,837	228,046	84.8%	35.3	3.1	−32.2
X	HepG2.2.15	319,139	299,509	93.9%	30.2	2.7	−27.6

^1^nucleotide length of primary deletions (i.e. reference segments not concordantly aligned to individual reads).

^2^nucleotide length of heterologous insertions observed at indel sites.

^3^net nucleotide gain/loss observed at indel sites.
