# Supplementary material for: CRISPR/Cas9 nickase-mediated disruption of hepatitis B virus open reading frame S and X
Source: Sci Rep. 2015 Sep 3;5:13734. doi: 10.1038/srep13734 (PMC4558539; doi:10.1038/srep13734)
Supplement: Supplementary Information [file srep13734-s1.pdf]

Supplementary Information

CRISPR/Cas9 nickase-mediated disruption of hepatitis B virus open reading frame S and X

Madina Karimova, Niklas Beschorner, Werner Dammermann, Jan Chemnitz, Daniela Indenbirken, Jan-Hendrik Bockmann, Adam Grundhoff, Stefan Lüth, Frank Buchholz, Julian Schulze zur Wiesch & Joachim Hauber

Context of supplementary information

1. Supplementary Figure legends
2. Supplementary Figure 1
3. Supplementary Figure 2
4. Supplementary Figure 3
5. Supplementary Figure 4
6. Supplementary Figure 5
7. Supplementary Table 1

Supplementary Figure 1 HBV reporter construct. The reporter plasmid for detecting Cas9n nuclease activity on HBV S or X target sequences was integrated into the genome of HEK293 or HeLa cells using the PiggyBac system (System Biosciences Inc.) to generate stable cell lines containing HBV reporters. Cells cotransfected with PiggyBac-HBV reporters and transposase were selected on puromycin and assessed for constitutive RFP expression.

Supplementary Figure 2 Distribution of deletion and insertion lengths. Distribution among indel amplicon reads derived from S-specific sgRNA expressing (A) HepG2-H1.3 and (B) HepG2.2.15 cells. The total length of individual deletions (red, upper graphs in each panel) or insertions (green, lower graphs in each panel) is shown in bins with 5 nucleotide increments. The bar height represents the total number of observed reads (log scale) with deletions/insertions of the given length. The '0'-value column represents reads without deletions or insertions.

Supplementary Figure 3 Distribution of deletion and insertion lengths. Distribution among indel amplicon reads derived from X-specific sgRNA expressing (A) HepG2-H1.3 and (B) HepG2.2.15 cells, as in Supplementary Figure 3.

Supplementary Figure 4 Consensus sequence of ORF S of HBV genotype A. Nucleotides appearing among isolates at a frequency higher than 0.01% are indicated below the consensus sequence. These positions in the consensus are referred to as semi-conserved and used to calculate the conservation of gRNA+PAM sequences. S-specific sgRNAs (i.e. sgRNA-S1, sgRNA-S2) are boxed, PAM sequences are boxed in gray.

Supplementary Figure 5 Consensus sequence of ORF X of HBV genotype A. Nucleotides appearing among isolates at a frequency higher than 0.01% are indicated below the consensus sequence. These positions in the consensus are used to calculate the conservation of gRNA+PAM sequences as in Figure S1. X-specific sgRNAs (i.e. sgRNA-X1, sgRNA-X2) are boxed, PAM sequences are boxed in gray.

Supplementary Figure 1

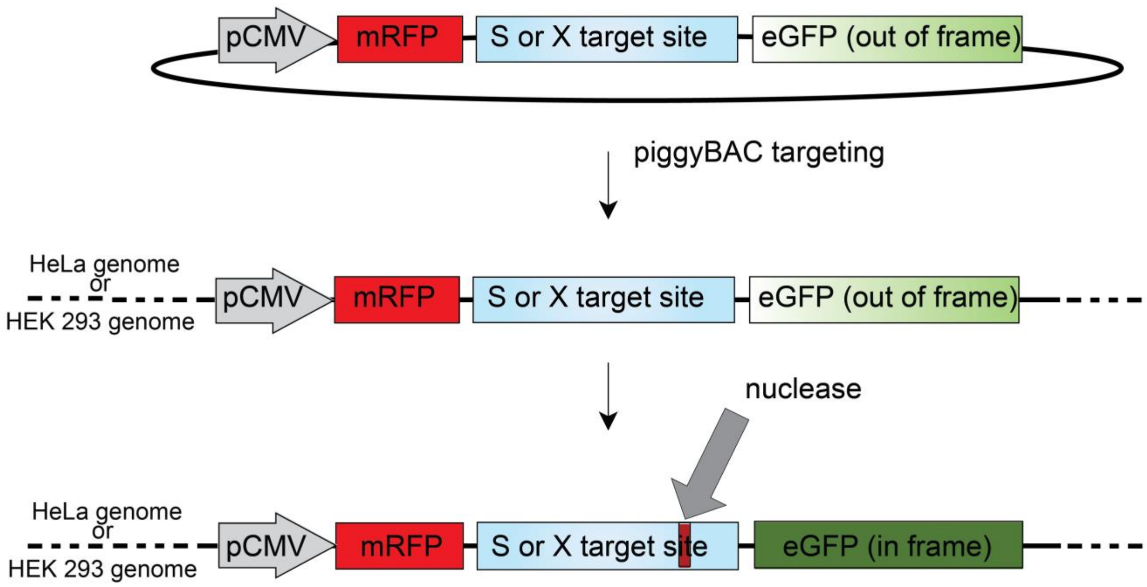

Supplementary Figure 2

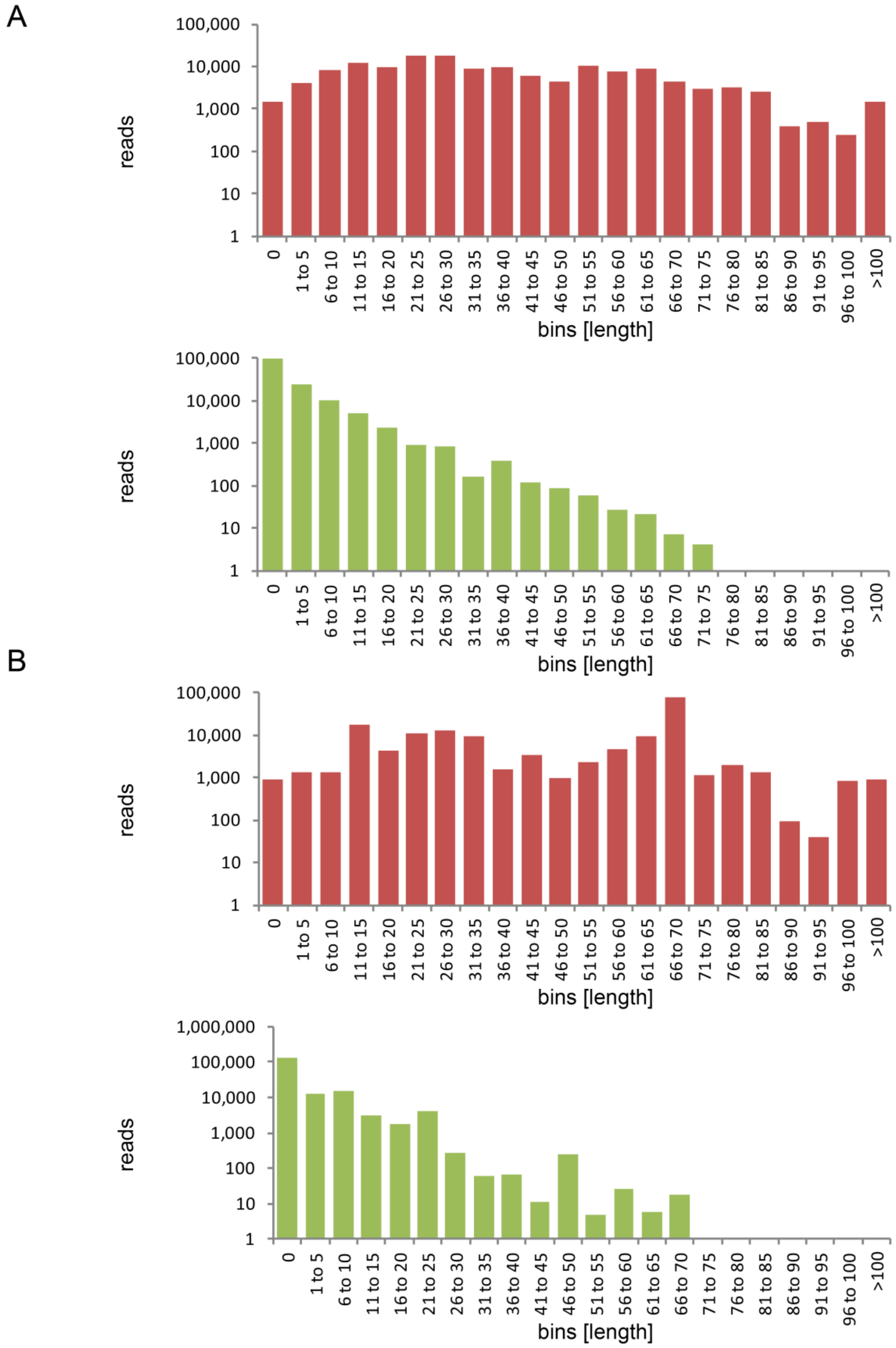

Supplementary Figure 3

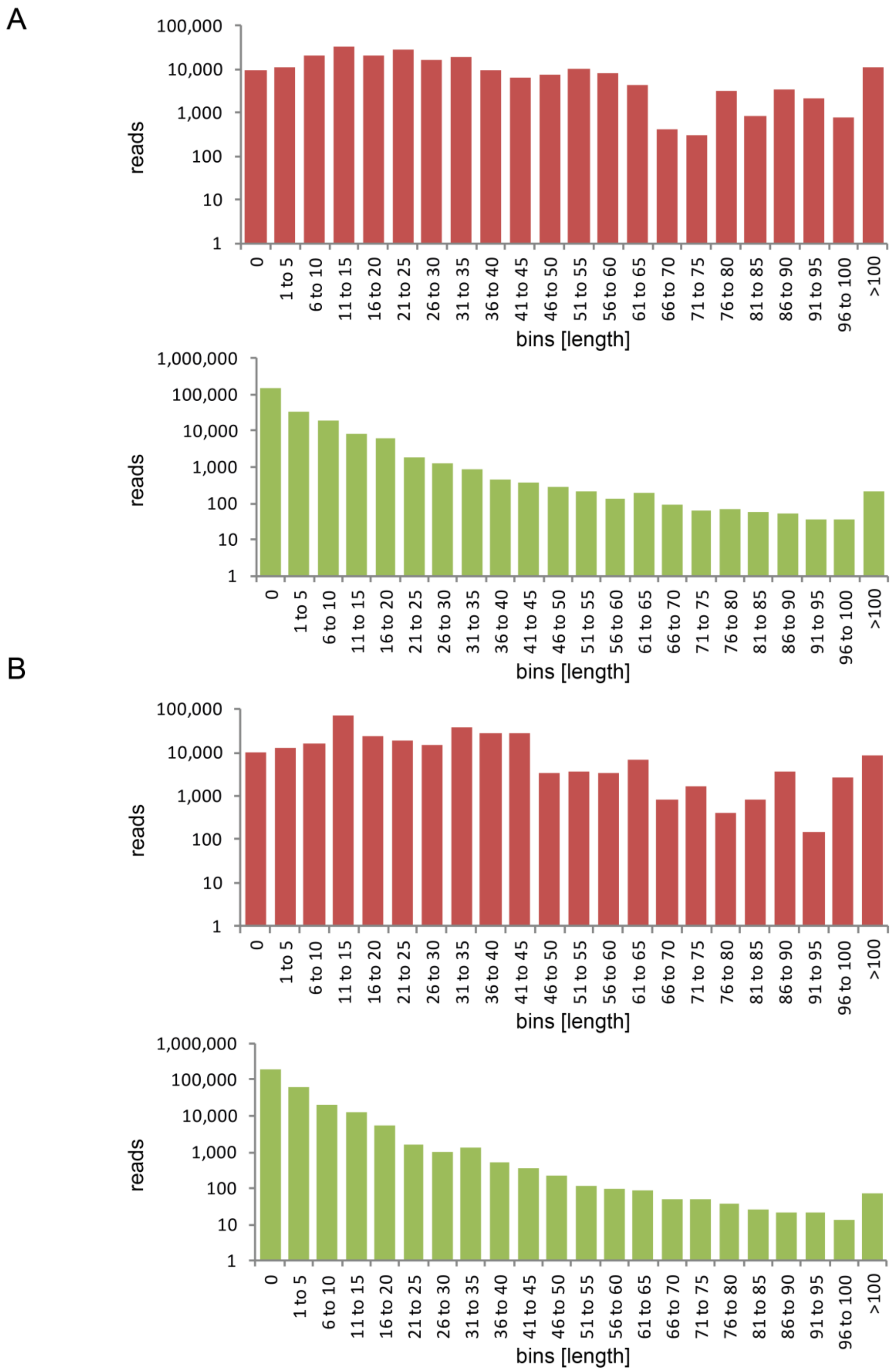

Supplementary Figure 4

```
Consensus sequence of HBV protein S

        10        20        30        40        50        60        70        80
....:....|....:....|....:....|....:....|....:....|....:....|....:....|....:....|
   *           *              *  *          **  *  *          *     ** * ***
ATGGAGAACATCACATCAGGATTCCTAGGACCCCTGCTCGTGTTACAGGCGGGGTTTTTCTTGTTGACAAGAATCCTCAC
     c               c     a                                          a

        90       100       110       120       130       140       150       160
....:....|....:....|....:....|....:....|....:....|....:....|....:....|....:....|
    *        *    *  *         *   **  *                      *     *  * *  *  *
AATACCGCAGAGTCTAGACTCGTGGTGGACTTCTCTCAATTTTCTAG-GGGGatCACCCGTGTGTCTTGGCCAAAATTCG
      a                               g            agg ta         g
                                                    cc
                                                     a

       170       180       190       200       210       220       230       240
....:....|....:....|....:....|....:....|....:....|....:....|....:....|....:....|
*    **        *** * **  *  * *      *  *  *   **    * *  *    * **     ** ** **
CAGTCCCCAACCTCCAATCACTCACCAACCTCCTGTCCTCCAATTTGTCCTGGTTATCGCTGGATGTGTCTGCGGCGTTT
                                t          c         c     t

       250       260       270       280       290       300       310       320
....:....|....:....|....:....|....:....|....:....|....:....|....:....|....:....|
*   *    **  * ** **  *     * *** * *      *    *        *      *     *        *
TATCATATTCCTCTTCATCCTGC---TGCTATGCCTCATCTTCTTATTGGTTCTTCTGGATTATCAAGGTATGTTGCCCG
 r    c                                      g        g       gc  g

       330       340       350       360       370       380       390       400
....:....|....:....|....:....|....:....|....:....|....:....|....:....|....:....|
                                                             **        *  *
TTTGTCCTCTAATTCCAGGATCa------ACAACAACCAGtACGGGACCaTGCAaAACCTGCACGACTCCTGCTCAAGGC
                      c      g g  c     c      a c    gg
                             t

       410       420       430       440       450       460       470       480
....:....|....:....|....:....|....:....|....:....|....:....|....:....|....:....|
*           *   *       *          *  *      *    * *           ***      *   *
AACTCTATGTTTCCCTCATGTTGCTGTACAAAACCTACGGAtGGAAATTGCACCTGTATTCCCATCCCATCgTCcTGGGC
         aa      c     t     c      tt  cc           t                 a  t
                                                     a

       490       500       510       520       530       540       550       560
....:....|....:....|....:....|....:....|....:....|....:....|....:....|....:....|
*  *  **  * **** ***     * *  ** *    * *      ***    **        *     *     *  *
TTTCGCAAAATACCTATGGGAGTGGGCCTCAGTCCGTTTCTCtTGGCTCAGTTTACTAGTGCCATTTGTTCAGTGGTTCG
           t        g           c         a
                                          c

       570       580       590       600       610       620       630       640
....:....|....:....|....:....|....:....|....:....|....:....|....:....|....:....|
           * *       **  ** *   *     * * *   **     *        *       *  *
TAGGGCTTTCCCCCACTGTTTGGCTTTCAGcTATaTGGATGATGTGGTAtTGGGGGCCAAGTCTGTACAgCATCgTGAGt
c              t   c       t  t   g t      a t   c          aa   ct  a  c t   ag
                                           t c              r                  k

       650       660       670       680       690
....:....|....:....|....:....|....:....|....:....|.
CCCTTTATACCGCTGTTACCAATTTTCTTTTGTCTcTGGGTATACATTTAA
      t            t         a t   t    c     ac---
```

Supplementary Figure 5

```
Consensus sequence of HBV protein X

        10        20        30        40        50        60        70        80
....:....|....:....|....:....|....:....|....:....|....:....|....:....|....:....|
        *  * *** ** **** * *** ** *  ** ** ** ** ** * **  ** **  ** * ****   *
ATGGCTGCTAGGcTGTACTGCCAACTGGATcCTTCGCGGGACGTCCTTTGTTTACGTCCCGTCGGCGCTGAATCCCGCGG
            t   g             t        a           c     t     a           t
            g

        90       100       110       120       130       140       150       160
....:....|....:....|....:....|....:....|....:....|....:....|....:....|....:....|
** * ** **  * *  *   **      *  **
ACGACCCCTCtCGGGGCCGCTTGGGaCTCTcTCGTCCCCTTCTCCGTCTGCCGTtCCagCCGACCACGGGGCGCACCTCT
       t  ga a  t        g  g a                       a  gt
          a  c           c                                a
          c

       170       180       190       200       210       220       230       240
....:....|....:....|....:....|....:....|....:....|....:....|....:....|....:....|
CTTTACGCGGTCTCCCCGTCTGTGCCTTCTCATCTGCCGGtCCGTGTGCACTTCGCTTCACCTCTGC-------------
          a                             a

       250       260       270       280       290       300       310       320
....:....|....:....|....:....|....:....|....:....|....:....|....:....|....:....|
---------------------------------------------------------------------------ACGTT

       330       340       350       360       370       380       390       400
....:....|....:....|....:....|....:....|....:....|....:....|....:....|....:....|
GCATGGAGACCACCGTGAACGCCCATCAGatCcTGCCCAAGGTCTTAcATAAGAGGACTCTTGGACTCcCAGCAATGTCA
      ca            nnnnnnnnnnnnnnnnnnnnnnnnnnnnnnnnnnnnnnnnnnnnnnnnnnnnnnnnnnnn
                         c  agg t              t     c              t

       410       420       430       440       450       460       470       480
....:....|....:....|....:....|....:....|....:....|....:....|....:....|....:....|
ACGACCGACCTTGAGGCcTACTTCAAA-----------------------------------------------------
nnnnnnnnnnnnnnnnnnnnnnnnnnnnnnnnnnnnnnnnnnnnnnnnnnnnnnnnnnnnnnnnnnnnnnnnnnnnnnnn
                 t

       490       500       510       520       530       540       550       560
....:....|....:....|....:....|....:....|....:....|....:....|....:....|....:....|
                                                                         *
-----------------------------------------------------------GACTGTGTGTTTAAgGACTGG
nnnnnnnnnnnnnnnnnnnnnnnnnnnnnnnnnnnnnnnnnnnnnnnnnnnnnnnnnnnnnnnnnnnacc   a------
                                                               a         -

       570       580       590       600       610       620       630       640
....:....|....:....|....:....|....:....|....:....|....:....|....:....|....:....|
GAGGAGcTGGGGGAGGAGAtTAGGTTAAaGgTc---TtTGTATTAGGAGGCTGTAGGCAtAAATTGGTCTGcgCACCAgc
------t---------------------t-a--   -----------------------g------------------t-
     a-            c    c   - - t    a                     -           tt     -t
                   a                                                          nn

       650       660
....:....|....:....|....:...
acCaTGCAACTTTTTCACCTCTGCCTAA
-t--------------------------
c-ncnnnnn      nn
nn n
t
```

Supplementary Table 1 Conservation of target sites. Upper panel: Percentage conservation of the 23 nt (20 nt complementary to gRNA plus 3 nt PAM sequence) in sgRNA/Cas9 target sequences S1, S2, X1 and X2 among the HBV genotypes B, C, D, E, F and G. Lower panel: DNA sequences of the gRNA binding sites in the conserved HBV regions. PAM motifs are oriented on the right-hand side and are highlighted by grey boxes. Positions with non-conserved nucleotides are indicated in italic. Non-conserved nucleotides appearing with a frequency of more than 1% are indicated underneath in lower case.

Conservation of 23 nucleotides sequence (20 gRNA + 3 PAM) in HBV genotypes, %				
	ORF S		ORF X	
genotype	S1	S2	X1	X2
A	100.0	95.3	100.0	100.0
B	95.9	98.1	97.9	93.3
C	97.5	98.4	96.9	98.4
D	89.1	95.5	100.0	100.0
E	99.0	98.8	95.0	98.0
F	low	low	low	low
G	93.7	90.3	97.7	62.0
H	low	low	low	low

HBV-S1 ACCCCGCCTGTAACACGAGC | AGG

HBV-S2 TACC*G*CAGAGTCTAGACTCG | TGG
a

HBV-X1 GTCTGTGCCTTCTCATCTGC | CGG

HBV-X2 GTAAAGAGAGGTGCGCCCCG | TGG
